# RNA binding motif protein 3: a potential biomarker in cancer and therapeutic target in neuroprotection

**DOI:** 10.18632/oncotarget.14755

**Published:** 2017-01-19

**Authors:** Ren-Bin Zhou, Xiao-Li Lu, Chen-Yan Zhang, Da-Chuan Yin

**Affiliations:** ^1^ Key Laboratory for Space Bioscience & Biotechnology, School of Life Sciences, Northwestern Polytechnical University, Xi’an, Shaanxi, PR China

**Keywords:** RNA binding protein 3, cancer, biomarker, neuroprotection

## Abstract

RNA binding motif 3 (RBM3) is a highly conserved cold-induced RNA binding protein that is transcriptionally up-regulated in response to harsh stresses. Featured as RNA binding protein, RBM3 is involved in mRNA biogenesis as well as stimulating protein synthesis, promoting proliferation and exerting anti-apoptotic functions. Nowadays, accumulating immunohistochemically studies have suggested RBM3 function as a proto-oncogene that is associated with tumor progression and metastasis in various cancers. Moreover, emerging evidences have also indicated that RBM3 is equally effective in neuroprotection. In the present review, we provide an overview of current knowledge concerning the role of RBM3 in various cancers and neuroprotection. Additionally, its potential roles as a promising diagnostic marker for cancer and a possible therapeutic target for neuro-related diseases are discussed.

## INTRODUCTION

RNA binding proteins (RBPs) or RNA binding motifs are involved in RNA metabolism at the post-transcription level by directly binding to double or single stranded RNA or messenger RNAs (mRNAs), which are essential in various cellular processes [1–4]. Among these, RNA binding motif 3 (RBM3) is originally discovered to be a cold shock response protein, which is high-expressed during hypothermia to possibly increase cell survival. Also, RBM3 is essential for cell proliferation and protects against adverse condition-induced cell death, such as hypothermia [5], hyperthermia [6], hypoxia [7], serum deprivation [8], radiation [9] as well as toxins and drugs [10]. Further studies suggested that RBM3 has cytoprotective functions and RBM3 overexpression can rescue polyglutamine-induced cell death in both non-neural cells and neural cells [11]. Indeed, expression of RBM3 is required for appropriate cell cycle progression and preventing mitotic catastrophe by increasing mRNA stability translation of rapidly degraded transcripts [12]. The G2-phase control role of RBM3 was uncovered in RBM3 silenced A2780 cells [13] and RBM3 knockout mice [14]. What's more, the significant increasing expression of RBM3 in old muscle revealed that it shows the ability to maintain or restore muscle mass during prolonged periods of disuse [15].

Apart from the functions mentioned above, accumulating evidences have suggested an essential role of RBM3 in cancers and neurodegenerative diseases. Therefore, in this review, we mainly focus on the functions of RBM3 in cancers and neurodegenerative diseases. We began with the structure, evolution and dynamically distribution of RBM3. Then, the roles of RBM3 in cancers and neuroprotection as well as the underlying mechanisms are discussed. At the same time, its potential roles as a promising diagnostic marker for cancer and a possible therapeutic target for neuro-relative diseases are outlined. Finally, the future perspectives of RBM3 are described.

## STRUCTURE, EVOLUTION AND EXPRESSION PATTERN

**Figure 1:**
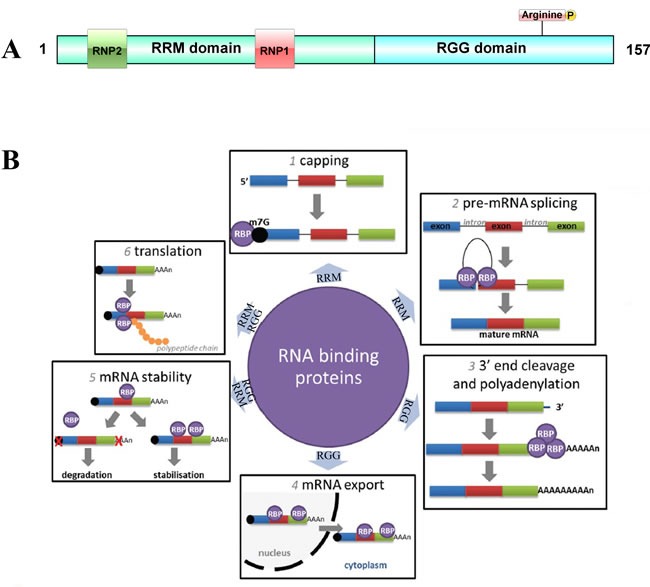
The posttranscriptional regulation controlled by RNA binding proteins **A**. The RRM domain and RGG domain of RBM3 are shown. **B**. The posttranscriptional regulation controlled by RNA binding proteins. Reprinted with permission from [18]

Human RBM3 was initially identified in fetal brain cDNA and mapped to Xp11.23. The open reading frame (ORF) sequence of the RBM3 gene encodes a 157-amino acid protein with a predicted molecular weight of 17 kD [16, 17]. Its structure is characterized by two highly conserved RNA recognition motif (RRM), namely RNP1 and RNP2 at the N-terminus and a less conserved arginine-glycine-rich domain (RGG) at the C-terminus (Figure 1A). Like other RBPs, the two domains exert their multifunction by controlling gene expression. RBPs control the gene expression steps mainly at the posttranscriptional level (Figure 1B) [18]. The RRM domain is involved in capping, it has demonstrated that cap-binding complex contains a classical RRM domain to bind RNAs [19]. The RRM domain is also critical for pre-mRNA splicing [20]. While, the RGG domain is mainly regulating cleavage and polyadenylation [21]. At the same time, the RGG domain, especially the arginine residue of the RGG domain, is essential for mRNA export. The absence of a single arginine residue in RGG domain interrupts nuclear-cytoplasmic shuttling [22]. Both the RRM domain and RGG domain is associated with mRNA stability and translation. The cold-induced RNA binding protein (CIRBP) can bind the 3′-UTR of thioredoxin (TRX) mRNA to stabilize its structure and promote its translation by RRM domain and RGG domain independently, and both domains are required for maximal binding [23].

**Figure 2:**
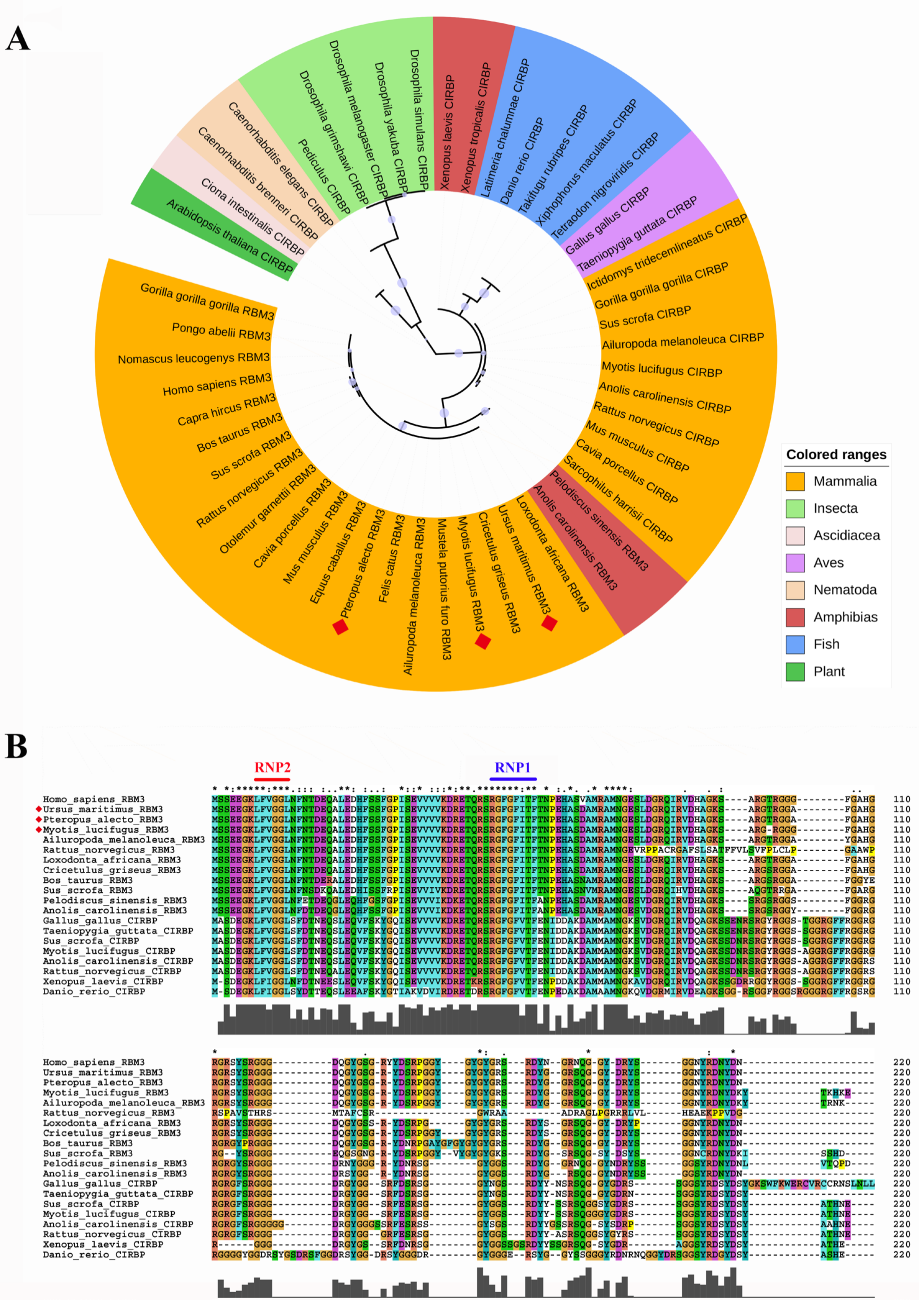
Phylogenetic relationship and multiple sequence alignment of human RBM3 and CIRBP in different species **A**. The evolutionary tree of RBM3 and CIRBP. Phylogenetic tree was built using online server tool iTOL [24]. Protein sequence was obtained by human RBM3 search in TreeFam database [25]. **B**. Multiple sequence alignment of human RBM3 and CIRBP. The conserved RNP2 and RNP1 motifs are highlighted in red and blue, respectively. The hibernating animals is marked with red diamond.

The evolution of RBM3 is conserved across animals, especially in mammalia, they belong to the same clade, as can be seen in Figure 2A, which shows the evolutionary tree of RBM3 and CIRBP (built using online server tool iTOL [24], data obtained from TreeFam database [25]). While, CIRBP, the homologue protein of RBM3, is widely existed in insecta, amphibia, fish, aves and plant, with diversity of phylogenetic lines. Multiple sequence alignment shows that both of them contain the conserved RNP2 and RNP1 motifs (Figure 2B). Notably, the two motifs are the same among different mammalia (including hibernating animals, marked with red diamond), which means the preservation of biological functions of RBM3 in mammalia.

**Figure 3:**
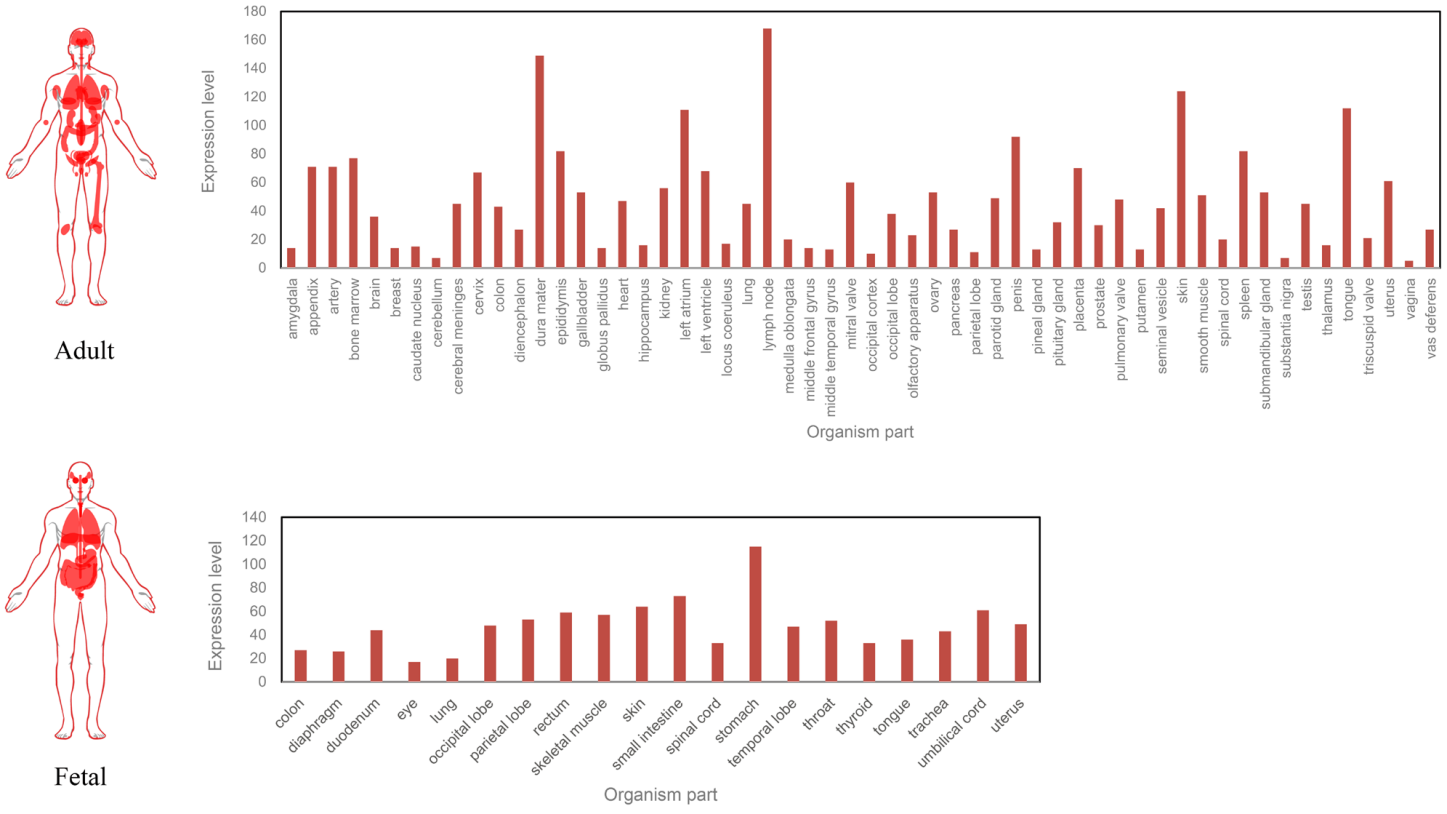
The expression pattern of RBM3 for different organism parts in “normal” condition between adult and fetal, data from Expression Atlas [27]

The spatial and spatiotemporal patterns of RBM3 are dynamically regulated. Except for its high expression in stress responses, RBM3 functioned as a key factor is also high expression during cell development. In rat brain, the expression level of RBM3 peaks during the first week postnatal in most brain areas and tapers off in youth and adulthood [26]. Alternatively, in newly formed and migrating neurons, high RBM3 expression is also observed [22]. Surprisingly, after searching the expression pattern of RBM3 for different organism parts in “normal” condition between adult and fetal in human from Expression Atlas [27](Figure 3), it was found that RBM3 is also abundant in adult, which suggested that RBM3 not only contributes to cell proliferation, differentiation, and plasticity during develop stages, but also plays an essential role in whole life.

## RBM3 IN CANCER

### RBM3 expression in cancer

Mounting immunohistochemical studies have demonstrated that RBM3 has proto-oncogenic potential because its expression is up-regulated in various human tumors and high RBM3 expression levels are associated with good prognosis in the clinic. Therefore, this protein is a potentially useful biomarker for cancer treatment. An immunohistochemistry (IHC) evaluation of RBM3 expression in various cancers is listed in Table 1.

**Table 1:** RBM3 expression in cancers

Tumor type	Quantification method (IHC)	Statistical analysis	Expression level compared with normal counterparts	Prognosis with high expression	Ref.
Colorectal cancer	Qualitative: nuclear staining intensity was recorded as 1+, 2+, or 3+	Staining results were categorized into three groups: negative, weak positive, and strong positive	Down-regulated	Good	[29]
Colorectal cancer	Semi-quantitative: 4 nuclear fraction groups: 0 (0-1%), 1 (2–25%), 2(26–75) and 3 (>75%). Nuclear staining intensity: 0=negative, 1=intermediate and 2=moderate-strong	RBM3 staining was denoted as negative, intermediate and strong in the survival analysis	Up-regulated	Good	[30]
Testicular non-seminomatous germ cell tumors	Semi-quantitative: 4 nuclear fraction groups: 0 (0-1%), 1 (2–25%), 2(26–75) and 3 (>75%) Nuclear staining intensity: 0=negative, 1=mild, 2=intermediate and 3=strong	RBM3 expression (intensity x fraction) was dichotomized into weak vs. strong using classification and regression tree (CRT) analysis	Up-regulated	Good	[95]
Urothelial carcinoma	Semi-quantitatively: Nuclear staining intensity: 0 =negative, 1 = weak staining, 2 =moderate staining, and 3 = intense staining.	None	Up-regulated	Without clinical outcome	[32]
Esophageal and gastric adenocarcinomas	Qualitative: nuclear intensity as 0 (negative), 1 (weak), 2 (moderate) and 3 (strong).	The RBM3 nuclear score was dichotomized into low and high for the survival analysis	Up-regulated	Good	[96]
Prostate cancer	Qualitative: nuclear staining intensity was recorded as 1+, 2+, or 3+	None	Up-regulated	Poor	[49]
Prostate cancer	Semi-quantitative: 4 nuclear fraction groups: 0 (0-1%), 1 (2–25%), 2(26–50%) and 3 (51-75%), 4(>75%) Nuclear staining intensity: 0=negative, 1= weak, 2= moderate 3= strong intensity	RBM3 expression (intensity × fraction) was dichotomized into weak vs. strong	Up-regulated	Good	[35]
Astrocytoma	Semi-quantitative: nuclear staining intensity: negative (–), weakly positive (+), moderately positive (++), and strongly positive (+++)	None	Up-regulated	Poor	[36]
Urothelial bladder cancer	Semi-quantitative: 4 nuclear fraction groups: 0 (0-1%), 1 (2–25%), 2 (26–75%) and 3 (>75%) Nuclear staining intensity: 0=negative, 1=mild, 2=intermediate and 3=strong	RBM3 expression was trichotomized into negative, intermediate, and high (survival analyses) and dichotomized into negative vs. positive or negative-intermediate vs. high	Down-regulated in metastases compared with primary melanoma	Good	[37]
Malignant melanoma	Semi-quantitative: 4 nuclear fraction groups: 0 (0-1%), 1 (2–25%), 2 (26–75%) and 3 (>75%) Nuclear staining intensity: 0=negative, 1=mild, 2=intermediate and 3=strong	The RBM3 nuclear score was dichotomized into low vs. high.	Up-regulated in primary tumors; weak or absent in metastases	Good	[38]
Malignant melanoma	Qualitative: nuclear staining intensity: 0=negative, 1=mild, 2=intermediate and 3=strong	Samples with scores of 0, 1, and 2 were pooled together and compared with samples with a score of 3.	Up-regulated in primary tumors, weak or absent in metastases	Good	[34]
Epithelial ovarian cancer	Semi-quantitative: 4 nuclear fraction groups: 0 (0-1%), 1 (2–25%), 2 (26–75%) and 3 (>75%) Nuclear staining intensity: 0=negative, 1= intermediate, 2= moderate to strong intensity	RBM3 expression was dichotomized into low vs. high.	Up-regulated	Good	[39]
Epithelial ovarian cancer	Semi-quantitative: 4 the nuclear fraction groups: 0 (0-1%), 1 (2–25%), 2 (26–75%) and 3 (>75%) Nuclear staining intensity: 0=negative, 1= intermediate, 2= moderate to strong intensity	RBM3 expression was dichotomized into negative vs. any expression	Up-regulated	Good	[13]
Breast cancer	Semi-quantitative: 4 the nuclear and cytoplasmic staining fraction groups: 0 (0–1%), 1 (2–25%), 2 (26–75) to 3 (>75%). Staining intensities within the respective subcellular locations were noted as 0=negative, 1=weak, 2=moderate and 3=strong	The RBM3 nuclear score was dichotomized into <75% vs. >75 % positive nuclear staining	Up-regulated	Good	[40]

Colorectal cancer (CRC), which is the fourth most common malignant disease worldwide, remains the second most common cause of death by cancer in western countries [28]. Two independent studies concluded that the loss of RBM3 expression was an unfavorable marker in CRC [29], and that high expression of RBM3 was an independent marker for prolonged overall survival in CRC patients [30].

Testicular germ cell tumors (seminomatous and nonseminomatous) are the most common cancer types among young adult men. The high overall cure rate of cisplatin-based chemotherapy has shifted interest in this cancer to reduce treatment-related toxicity and increase positive prognoses [31]. A study focused on testicular non-seminomatous germ cell tumors (NSGCT) revealed that the risk of treatment failure in patients with metastatic NSGCT was increased when RBM3 was lowly expressed, which suggested that RBM3 was a potential predictor for treatment in patients with metastatic NSGCT [31].

The putative prognostic roles of RBM3 in both invasive and metastatic urothelial carcinoma (UCA) were also analyzed [32]. The results indicated that stronger RBM3 expression was correlated with UCA metastasis, but not with the clinical prognostic outcome.

In the upper digestive system, RBM3 is highly expressed in esophageal and gastric adenocarcinoma, and reduced RBM3 expression is an independent factor of reduced overall and recurrence-free survival in these patients. Compared with a normal oral epithelium, human papillomaviruses-oropharyngeal carcinomas exhibit reduced RBM3 expression, but a potential biomarker for this of type cancer should be further pursued [33].

Although RBM3 is overexpressed in prostate cancer, different prognostic results have been reported. One study suggested that high RBM3 expression was associated with a good prognosis [34], and another study showed that high RBM3 expression was an independent prognostic marker of early biochemical recurrence and disease progression [35]. These divergent results may be explained by the differences in experimental procedures, patient selection or cohort size.

The mRNA expression level assessment, western blotting and immunohistochemical staining results have demonstrated that RBM3 was up-regulated in human astrocytomas compared with normal brain tissues. Additionally, a higher astrocytoma grade suggested stronger RBM3 expression, indicating that RBM3 overexpression might have proliferative and/or proto-oncogenic functions in humans [36].

In urothelial bladder cancer (UBC), RBM3 expression is reduced in metastases compared with primary melanoma. Reduced RBM3 expression is significantly associated with more aggressive tumors and is an independent predictor of reduced survival in patients suffering from UBC [37]. Similar to UBC, reduced RBM3 expression is also observed in metastases in malignant melanoma compared with primary melanoma. Loss of RBM3 expression is associated with clinically more aggressive tumors and is an independent factor of poor prognosis in malignant melanoma [38].

In epithelial ovarian cancer (EOC), RBM3 overexpression is correlated with a favorable prognosis. Down-regulation of RBM3 confers reduced cisplatin sensitivity and is involved in DNA integrity and the cell cycle [13, 39]. In breast cancer, a leading cancer type in women, RBM3 is overexpressed and has been associated with favorable clinicopathological parameters in two breast cancer cohorts [40].

In summary, various immunohistochemical studies have demonstrated the consistent conclusion that high RBM3 expression is associated with a good prognosis. The only exceptions published to date include astrocytoma and radically operated prostate cancer, in which high RBM3 expression is associated with a higher grade. These findings have attracted attention to the characterization of the mechanisms of RBM3 in cancer.

### Mechanism of RBM3 in cancer

The roles of other RBPs in cancer have been elucidated [41–44], but the literature concerning the exact mechanisms of RBM3 in cancer are conflicting. For instance, RBM3 has been noted high expression in various cancers and then featured as a potential proto-oncogene. The underlying mechanisms follows, (1) RBM3 overexpression induces oncogenic transformation. When RBM3 overexpressed in NIH3T3 mouse fibroblast cells, there was a significantly higher level of cell proliferation and the cells formed tight, densely packed multi-cellular spheroids compared with the wild-type. Also, high expression RBM3 in the already transformed SW480 colon cancer cells resulted in larger colonies and higher levels of proliferation than control. In contrast, silencing RBM3 decreased HCT116 colon adenocarcinoma cell proliferation and a complete shut down of tumor xenograft growth [12]. The suitable explanation is that RBM3 enhances the mRNA stability and translation of some tumorigenesis targets, such as cyclooxygenase 2(COX-2), interleukin (IL-8) and vascular endothelial growth factor (VEGF) [12]. (2) RBM3 is indispensable for cell cycle progression. High level RBM3 protects cancer cells from mitotic catastrophe [12] or apoptosis [45]. Silencing RBM3 expression by siRNA arrests cells in the G2/M phase and results in cell apoptosis [13]. (3) High RBM3 levels increase stem cell characteristics. It has been assessed in colorectal cancer cells by measuring stem cell features, such as the side population, spheroid formation capacity and stem cell markers. The partial mechanism for RBM3 overexpression may involve in increasing β-catenin signaling, a pathway that enhances cancer stemness [46]. High RBM3 levels suppresse GSK3β activity by phosphorylation at Ser9. This modification leads to an inability to degrade β-catenin and results in nuclear accumulation of β-catenin, thus activating the β-catenin signaling pathway [47]. However, another study suggested that high RBM3 attenuated the stemness and tumorigenesis by inhibiting CD44 variant splicing in prostate cancer cells [48]. The possible mechanism proposed by the authors is that RBM3 in cancers involves in enhancing the balance between CD44v and CD44s expression to attenuate the stem cell like features to adapt to the change in the microenvironment for survival and repopulation of tumor cells. This phenomenon explains how the increased expression of RBM3 in the early stage of tumor cells promotes tumor development (high rate of cell proliferation and protein synthesis), whereas its downregulation in subsequent steps is essential for tumor progression (migration, invasion, and recolonization). (4) High RBM3 expression is an independent predictor of poor prognosis. RBM3 has been shown to be high-expressed in poorly differentiated, more aggressive prostate cancer and an independent prognostic factor predicting early recurrence [49, 50]. At the same time, RBM3 high expression has been shown to be correlated with high grade astrocytoma compared with low grade or normal tissue [36].

**Figure 4:**
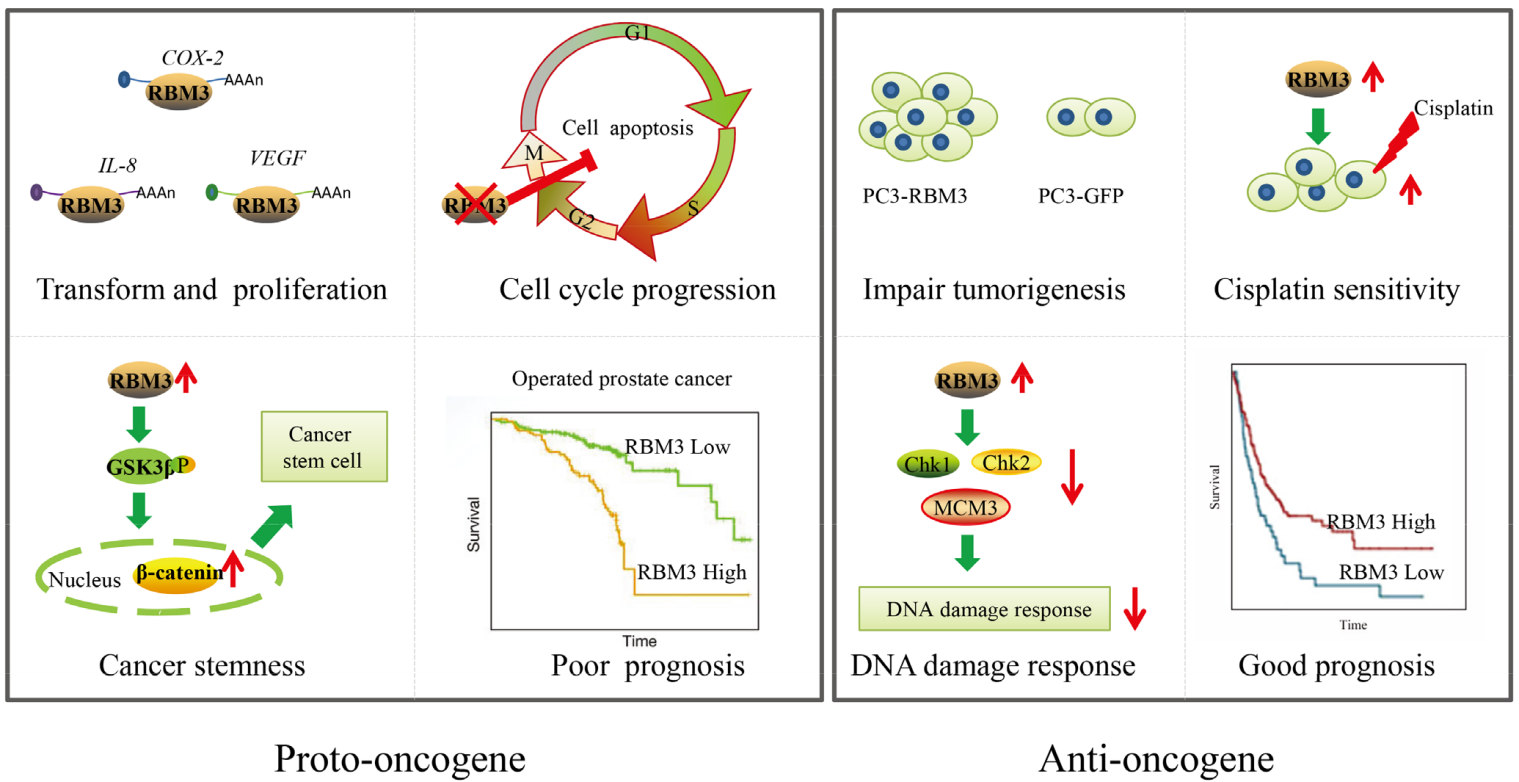
The possible roles of RBM3 as proto-oncogene or anti-oncogene

Conversely, RBM3 is also associated with inhibition of tumor growth and dissemination. It has demonstrated high expression of RBM3 is correlated with decreasing tumor progression, recurrence and increasing disease free survival in breast cancer [40]. In melanoma, down-regulated RBM3 was observed in metastases compared to primary melanoma, in line with previous *in vitro* data down-regulated RBM3 in metastatic compared to primary melanoma cells, the low RBM3 tumors correlated with increased aggressiveness [34]. RBM3 overexpression impairs tumorigenesis in PC3 cells, it has demonstrated that in PC3-RBM3 cells the tumor volume was smaller or no tumor found compared with control PC3-GFP cells [48]. What's more, high RBM3 increased cisplatin sensitivity of cancer cells. RBM3 levels were higher in cisplatin-sensitive than cisplatin-resistant ovarian cancer cells [13]. Last but not least, the clinical studies have shown that high RBM3 is associated with a better prognosis in most cancers listed in Table 1. The mechanism of RBM3 in the DNA damage response is a reasonable explanation [39]. It is hypothesized that high RBM3 expression indirectly contributes to low levels of some checkpoint proteins, such as DNA damage checkpoint kinases (CHK1 and CHK2) and minichromosome maintenance protein 3 (MCM3). Silencing RBM3 expression resulted in an obvious increment in Chk1, Chk2 and MCM3 in A2780 cells [39]. In line with RBM3 down-regulated led to activation of both Chk1 and Chk2 in colorectal cancer cell lines [12]. This finding partly explains the previously demonstrated effect of RBM3 on cisplatin sensitivity (inhibition of CHK1 exhibits a great impact on cisplatin response in ovarian cancer cells [51]) and that RBM3 overexpression is associated with a good prognosis in various cancers, because the invasion and metastatic spread of cancers may be inhibited by a deficient DNA repair system. The possible roles of RBM3 as proto-oncogene or anti-oncogene are summarized in Figure 4.

Importantly, the role of RBM3 in different cancers remains contested. Cancer is a complex genetic disease and many regulatory factors involved in this process. Specific RBPs can control the expression of numerous onco-proteins or tumor suppressors and those mRNAs are highly regulated by splicing, stability, localization as well as translation in a tissue-specific manner [43]. Thus, it can be assumed that the exact role of RBM3 in cancers is largely dependent on the cancer type and the molecular context activated in different pathways. The prognostic impact of the loss of RBM3 expression is markedly pronounced in estrogen receptor (ER)-positive breast cancer compared with ER-negative tumors [40], whereas RBM3 overexpression is a good prognostic marker in prostate cancer, which is governed by androgen receptor (AR) signaling [35]. RBM3 also exhibits neuroprotection functions in nerve cells, so the associated protein activation in astrocytoma may be not essential in non-neuronal cell. In epithelial ovarian cancer, the mechanism is associated with the inhibition of MCM3, Chk1 and Chk2 [39]. By contrast, in prostate cancer, the mechanism is involved in the activation of ERG, depletion of PTEN [49], and CD44 variant splicing [48]. In colorectal cancers, RBM3 enhances Wnt/β-catenin signaling mediated by inactivation of GSK3β [47]. *In vitro* analysis revealed that RBM3 overexpression increased cell proliferation in SW480 human colon epithelial cells, and the process is associated with COX2, VEGF and cyclin D1 [12]. In melanoma, RBM3 is involved in the inhibition of MCM3 [38]. In breast cancer, the target gene is Bax [40]. RBM3 knockdown was more effective in LNCaP cells compared with PC-3 cells, suggesting that at least a component of RBM3 function may be cell type-dependent. Another RNA binding protein HuR, it has demonstrated that HuR targets mRNAs including COX2, oncogenes, cyclins, cyclin-dependent kinases, and contributes to tumorigenesis. Additionally, HuR is also responsible for tight regulation of tumor suppressor p21 and Wnt family protein Went-5a, indicating its role in tumor suppression [52]. The exact RBM3 pathway involved requires further investigation.

### RBM3 as a valuable cancer biomarker

Despite the advancements in current cancer therapies, including surgical resection, chemotherapy, radiotherapy and combination immunotherapy, the incidence of cancer is constantly increasing worldwide [53]. Additionally, the lack of accurate prognostic biomarkers results in overtreatment or inadequate treatment of cancer patients [54]. Thus, a new biomarker with better stratification of cancer patients into different risk categories and prognostic value is urgently needed. Different expression levels of RBM3 have a significant connection to patient survival in various cancers, tremendous efforts have focused on studying the diagnostic and prognostic value of RBM3. The Human Protein Atlas (HPA) project (proteinatlas.org), an excellent tool for new biomarker discovery, has already featured RBM3 as a potential biomarker in cancers [55]. The high expression of RBM3, which is associated with improved patient survival and cisplatin sensitivity, may help to predict tumor behavior and guide optimized personalized therapy in individuals (Table 2).

**Table 2:** RBM3 as biomarker in different cancers

Cancer type	Method	Clinical use	Adjuvant therapy
Breast cancer	IHC	Stratify Monitor	High RMB3: less intense Low RMB3: more aggressive
Ovarian cancer	IHC	Stratify	For stage III-IV patients platinum or cisplatin treatment high RBM3: need
Malignant melanoma	IHC	Stratify Monitor	High RMB3: less intense Low RMB3: more aggressive
Colon cancer	IHC	Stratify	For stage II patients High RMB3: not necessary Low RMB3: necessary
Urothelial cancer	IHC	Stratify Monitor	For stage T1 patients High RMB3: needn't cystectomy Low RMB3: need cystectomy

In breast cancer, surgery is the primary curative treatment and the adjuvant therapy is need for advanced patients. However, today the major clinic need is reducing the overtreatment. High RBM3 expression was associated with small, low-grade, ER-positive cancers, and improvement in recurrence free survival. It indicated that a less intense treatment for high-RMB3 patients, and more aggressive treatment for low-RMB3 patients in clinic.

In ovarian cancer, cisplatin treatment is a cornerstone, but side effects are severe. A guide decides whether receiving cisplatin treatment or treatment intensity mean an improvement in patient care. Studies revealed that RBM3 is higher expressed in cisplatin sensitive cells, and the RBM3 silenced cells became significantly less sensitive to cisplatin treatment. Thus high RBM3 expression patient received cisplatin treatment had a markedly increased survival compared with non-cisplatin based adjuvant treatment. The result was also verified in colorectal cancer.

Malignant melanoma tends to metastasize and no prognostic information to estimate the risk for metastatic disease. Studies showed that the probability of metastasis is about 50% in RBM3-low expression primary tumors, whereas in RBM3-high expression case, it is approximately 30%. Thus, detecting RBM3 expression can suggest physicians whether patients should be more closely monitored. It also suggested that a less intense treatment for high-RMB3 patients, and more aggressive treatment for low-RMB3 patients in clinic.

In colorectal cancer, patients with stage III and high-risk stage II disease recommended adjuvant treatment, However, not all high-risk stage II patients benefit from adjuvant treatment. The overall survival of high RBM3 stage II patients is higher than low RBM3 patient, indicating that high RBM3 stage II patient is not necessary to receive adjuvant treatment.

In urothelial cancer, due to high recurrence and progression into muscle invasive disease, the monitor is needed. It has demonstrated that RBM3 down-regulated in metastases compared with primary and RBM3 expression can be used to identify stage T1 patients whether need of cystectomy. 5-year overall survival suggested the low RBM3 expression T1 patients in need of cystectomy, while, RBM3 high expressed needn’t. At the same time, for other cancers, such as astrocytoma, testicular and prostate cancer, the different expression level of RBM3 can also give some useful information for aiding physicians in monitoring disease metastatic and stratifying patient for adequate adjuvant treatment. However, to date, there are no certified methods for RBM3 measurements in patient samples that can be quickly and easily conducted in the clinic. Moreover, possible false prognosis could be made due to other effectors of RBM3 regulation, i.e. hypoxia, hypothermia and UV radiation.

## RBM3 IN NEUROPROTECTION

### The role of RBM3 in neuroprotection

In addition to the roles of RBM3 in cancer, increasing evidence has described its neuroprotective functions, as demonstrated by five aspects.

First, RBM3 was initially identified in a fetal brain cDNA library. RBM3 is a key factor in the rat brain during early development, especially during the first to second postnatal weeks (mainly due to the increased numbers of neurons), in both humans and mice [26, 56]. RBM3 is also highly expressed in newly formed and migrating neurons and in the proliferative zones and high translation rate zones of the adult brain [22, 26]. The dynamic regulation of RBM3 in the brain indicates that RBM3 is essential for neural cell proliferation and differentiation. Besides, characterized as one of RBPs, RBM3 should be implicated in neuroprotective function similar to other RBPs [57–60].

Second, the neuroprotective role of RBM3 is related to cell survival *in vitro*. RBM3 overexpression inhibited polyglutamine-induced cell death in SK-N-SH neuronal cells [11] and promoted protein synthesis in mouse neuroblastoma N2a cells by binding the 60S ribosomal subunits and altering the microRNA levels [61]. Additionally, mild hypothermia-induced RBM3 expression rescued PC12 neuronal cells from apoptosis, and inhibition of RBM3 expression *via* specific siRNAs significantly diminished the neuroprotective effect [62]. The observed up-regulation of RBM3 mRNA and protein expression in murine hippocampal brain slices and HT-22 neuronal cells also supports a potential role for RBM3 in hypothermia-induced neuroprotection [63].

Third, hypothermia-mediated neuroprotection has been successfully applied in the clinic for neuroprotection following cardiac arrest, stroke and traumatic brain injury [59, 64]. Interestingly, during hibernation and hypothermia, global protein synthesis and cell metabolism are down-regulated, whereas RBM3 is highly expressed. RBM3 seems to be related to hypothermia-mediated neuroprotection. Clinically relevant moderate (33.5°C) hypothermia treatment leads to significant RBM3 mRNA and protein up-regulation in murine organo-typic hippocampal slice culture (a suitable tissue culture system to investigate various aspect of neuronal-protection and function *in vitro*) [63]. Hypothermia is also associated with protecting neuron stem cells in the dentate gyrus (DG) region [65]. RBM3 was dramatically up-regulated in the DG region in cultured hippocampal slices [66]. During hypoxic neuronal injury, RBM3 exerts its neuroprotective functions though a mechanism that does not involve hypoxia inducible factor 1 (HIF-1) [7]. These results support RBM3 as a potential effector of hypothermia-induced neuroprotection.

Fourth, RBM3 also plays a pivotal role in spinal cord injury (SCI), which is an important health problems worldwide. In response to SCI, RBM3 expression was significantly up-regulated in neurons and astrocytes at 1 day after SCI compared with the controls (sham spinal cord injury) [67]. Coincidently, in the same SCI model, neuronal apoptosis and astrocyte proliferation peaked on the first day post-injury [68], suggesting that RBM3 was induced after SCI and was associated with an important neuroprotective function. Consistent with this finding, additional research uncovered the neuroprotective functions of RBM3 in an SCI rat model [69].

Finally, in neurodegenerative diseases, RBM3 exerts a neuroprotective function *via* structural plasticity, a process wherein synapses are continuously remodeled by dismantling and reassembling processes in healthy adult brain. Peretti et al. revealed that RBM3 overexpression increased by either boosting endogenous levels through hypothermia or *via* lentiviral delivery sustained synaptic protection, prevented behavioral deficits and neuronal loss and significantly prolonged survival in prion-infected and 5XFAD mouse models. In contrast, silencing RBM3 expression by knockdown or siRNA results in exacerbating synapse loss, accelerating disease and inhibition of the neuroprotective effects in both models. Moreover, RBM3 overexpression alone in the absence of cooling results in a similar neuroprotective effect. In conclusion, early synapse loss in mouse models of neurodegenerative diseases that results from defective synaptic repair processes is associated with failure to induce RBM3 protein expression. These results indicate that RBM3 may yield insights into endogenous repair processes and offer new therapeutic targets for neuroprotection in neurodegenerative diseases [70]. Furthermore, synaptic transmission is ameliorated to save energy in hibernating mammals. Thus, the synapses are dismantled in the hibernators’ brains, and are reassembled upon warming, which is also a form of structural plasticity. High RBM3 expression in hibernating mammals is additional evidence supporting its neuroprotective function [71, 72].

### Mechanism of RBM3 in neuroprotection

The mechanism of RBM3 in neuroprotection has not been characterized to date. Possible molecular pathways of RBM3 can enhance neural cell transcription and translation to maintain neuron stem cell numbers and functions [41]. Global protein synthesis is inhibited under stresses, such as hypothermia and hypoxia [73]. RBM3 expression is up-regulated and can enhance various aspects of global translation, including (1) stabilizing mRNA structures or acting as chaperones to stimulate nuclear-cytoplasmic transport [22]; (2) independently binding to the 60S ribosomal subunits on RNA [61]; (3) stimulating the activation of protein translation initiation (eukaryotic initiation factor 4E); (4) dephosphorylating eukaryotic initiation factor 2 alpha (eIF2α) and activating polysome formation [22]; and (5) altering miRNA levels [61, 74]. Mounting evidence suggests that RBM3 enhances cellular proliferation and differentiation by promoting or inhibiting specific miRNA formation. More than 60% of detectable miRNAs were significantly decreased in a neuronal cell line when RBM3 was silenced [74]. Additionally, the biogenesis of all members of the let-7 family, which are implicated in neural differentiation was greatly enhanced by RBM3 [75, 76].

**Figure 5:**
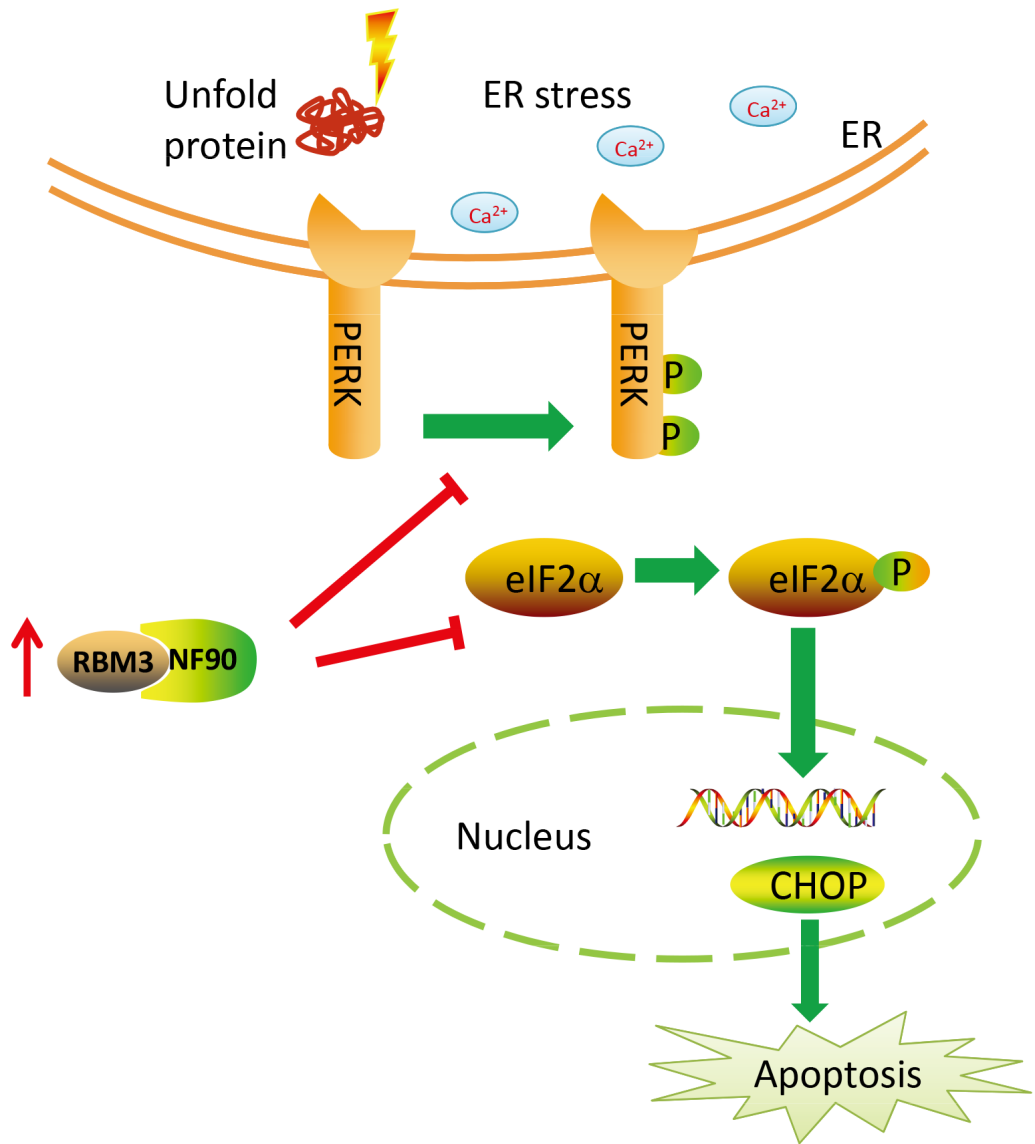
Model of the RBM3 neuroprotective function by inhibiting the PERK-eIF2α-CHOP ER stress pathway RBM3 acts as a PERK inhibitor in a NF90-dependent manner. RBM3 hinders the PERK-eIF2α-CHOP ER stress pathway and rescues cell apoptosis.

RBM3 up-regulation may also rescue neuronal cells from apoptosis [77, 78]. Endoplasmic reticulum (ER) stress improves neurological deficits and reduces cell apoptosis [79]. The ER is a critical organelle involved in protein synthesis/folding and calcium homeostasis [80]. When disturbed by various physiological and pathological insults, misfolded or unfolded proteins accumulate in the ER in a condition referred to as ER stress. ER stress subsequently triggers other intracellular signaling pathways as part of the unfolded protein response (UPR) [81]. The PERK-eIF2α-CHOP signaling pathway is one of three canonical signaling pathways involved in UPR activation. RBM3 exerts its cell-protective effects by modulating canonical PERK-eIF2α-CHOP signaling [82]. Under sustained ER stress, PERK and eIF2α phosphorylation is inhibited in cells overexpressing recombinant RBM3, which reduces CHOP expression and ultimately prevents cell apoptosis [83]. In contrast, PERK and eIF2α phosphorylation is dramatically increased in cells with silenced RBM3 by specific siRNAs, indicating that RBM3 may function as an inhibitor of the PERK-eIF2α-CHOP signaling pathway. Specifically, RBM3 inhibits PERK phosphorylation in a nuclear factor 90 (NF90)-dependent manner, ultimately reducing apoptosis (Figure 5). Besides, the inhibited apoptosis role of high RBM3 is exerted by reducing cleavage of polymerase. It was found that found that RBM3 over-expression reduced cleavage of poly ADP-ribose polymerase (PARP) and then inhibits staurosporine-induced apoptosis in neuron-like PC12 cells [62].

To date, the mechanisms underlying the association of RBM3 with structural plasticity are poorly characterized, but recent findings suggest that RBM3 may play an essential role in restoring synaptic plasticity during hibernation and hypothermia. RBM3 overexpression prevents the loss of synapses and alleviates behavioral and memory impairments. In contrast, synapse loss is increased when RBM3 is knocked down by siRNA [84]. Alternatively, a feature of the neurodegenerative disorder is the accumulation of disease-specific misfolded proteins, which results in ER stress [85]. Thus, the structural plasticity of RBM3 is also involved in the PERK-eIF2α-CHOP ER stress pathway.

### RBM3 as neuroprotection target

As indicated in the previous sections, the neuroprotective function of RBM3 is dynamically regulated to enhance neural cell proliferation and brain development. *In vitro*, high RBM3 expression rescues neuronal cells from forced apoptosis, whereas inhibition of RBM3 expression in neuronal cells by specific siRNAs significantly diminishes the neuroprotective effect. In neurodegenerative diseases and brain injury models, high RBM3 expression is also involved in neuroprotection. hypothermia-mediated neuroprotection has been successfully applied in the clinic for neuroprotection following cardiac arrest [86, 87], stroke [88] and traumatic brain injury [89, 90], but the procedure is associated with complications (cardiovascular effects and metabolic and electrolyte changes) [91]. For example, long-term hypothermia-mediated neuroprotection is a risk in the treatment of psychiatric patients with organic brain damage and may increase hypothermia susceptibility to antipsychotics [92]. These limitations are also applicable to patients with Alzheimer's disease [93]. Fortunately, the emerging role of RBM3 in hypothermia-induced neuroprotection offers the potential to identify new drugs without cooling to achieve the same goal. A combination of RBM3 and local hypothermia or short-term hypothermia may be another strategy with a better outcome. Alternatively, RBM3 can be targeted as a diagnostic biomarker for patients with neurodegenerative diseases. The RBM3 levels in the patient's blood can also be assessed. With this important information, the physician can plan more precise treatment [94]. Additionally, this information can facilitate the work of researchers in neural-related disease fields.

## CONCLUSIONS AND PERSPECTIVES

In this manuscript, the multifunction of RBM3 in cancer and neuroprotection is reviewed through summarizing the current literature. RBM3 is found to elevate expression in different kinds of cancers and is connected with clinic outcome, then it suggested that RBM3 may be a potential favorable biomarker in clinical diagnoses. However, the expression level and clinical behavior of RBM3 in cancer is still conflicting, more regulation mechanisms of RBM3 in oncogenic or tumor suppressor are still in great need both *in vitro* and *in vivo*. Targeted as biomarker, there is a continuing need to assess the relationship of RBM3 expression level in different stages of various cancers to stratify patient for personalized prevention and therapy.

In neuroprotection, although RBM3 is a promising therapeutic candidate for neuroprotection in prion-infected and 5XFAD models, it is important to note that the research has only been performed in mice and only at an early stage. Considerable efforts should be made to determine whether the same RBM3-mediated neuroprotective effects occur in humans before drugs are developed to mimic the protective effects of cooling. The therapeutic implications of RBM3 in the future should target the RBM3 directly (mimic its function or regulate its expression) or the signaling pathways it involved in.
